# Oral dosing with papaya latex is an effective anthelmintic treatment for sheep infected with *Haemonchus contortus*

**DOI:** 10.1186/1756-3305-4-36

**Published:** 2011-03-15

**Authors:** David J Buttle, Jerzy M Behnke, Yvonne Bartley, Hany M Elsheikha, David J Bartley, Martin C Garnett, Alison A Donnan, Frank Jackson, Ann Lowe, Ian R Duce

**Affiliations:** 1 Department of Infection & Immunity, University of Sheffield Medical School, Sheffield, S10 2RX, UK; 2 School of Biology, University of Nottingham, University Park, Nottingham, NG7 2RD, UK; 3 Parasitology Division, Moredun Research Institute, Pentlands Science Park, Bush Loan, Penicuik, Midlothian, EH26 0PZ, UK; 4 School of Veterinary Medicine and Science, University of Nottingham, Sutton Bonington Campus, Leicestershire, LE12 5RD, UK; 5 School of Pharmacy, University of Nottingham, University Park, Nottingham, NG7 2RD, UK

## Abstract

**Background:**

The cysteine proteinases in papaya latex have been shown to have potent anthelmintic properties in monogastric hosts such as rodents, pigs and humans, but this has not been demonstrated in ruminants.

**Methods:**

In two experiments, sheep were infected concurrently with 5,000 infective larvae of *Haemonchus contortus *and 10,000 infective larvae of *Trichostrongylus colubriformis *and were then treated with the supernatant from a suspension of papaya latex from day 28 to day 32 post-infection. Faecal egg counts were monitored from a week before treatment until the end of the experiment and worm burdens were assessed on day 35 post-infection.

**Results:**

We found that the soluble fraction of papaya latex had a potent *in vivo *effect on the abomasal nematode *H. contortus*, but not on the small intestinal nematode *T. colubriformis*. This effect was dose-dependent and at tolerated levels of gavage with papaya latex (117 μmol of active papaya latex supernatant for 4 days), the *H. contortus *worm burdens were reduced by 98%. Repeated treatment, daily for 4 days, was more effective than a single dose, but efficacy was not enhanced by concurrent treatment with the antacid cimetidine.

**Conclusions:**

Our results provide support for the idea that cysteine proteinases derived from papaya latex may be developed into novel anthelmintics for the treatment of lumenal stages of gastro-intestinal nematode infections in sheep, particularly those parasitizing the abomasum.

## Background

The traditionally available broad-spectrum anthelmintics for treatment of gastro-intestinal nematode infections in ruminants are largely limited commercially to three classes of drugs, namely benzimidazoles, imidazothiazoles/tetrahydropyrimidines, and macrocyclic lactones, which have been in use extensively for some 30 to 50 years. New classes have been launched in the last few years [1,2], represented by emodepside, monepantel, and derquantel, but these drugs are fairly expensive and so far have very limited label claims for efficacy. For example, emodepside is labelled only for treatment of enteric worms in cats, and in combination with praziquantel tablets (Profender^®^) for dogs, and monepantel and derquantel (in combination with abamectin) are labelled only for use in sheep. Nonetheless, while it is likely that some of these drugs may be labelled for use in more species in the future, their cost will be much higher than products of the older anthelmintic classes. Thus, it appears most likely that the development of resistance will continue to outpace the introduction of new anthelmintic drugs, and any new drugs will be much more expensive. As a result, there is a need for more therapeutic agents in the market, because of the rapidity with which resistance develops in nematodes when anthelmintics are applied intensively under typical large flock industrial-scale husbandry conditions [3,4].

Natural plant-derived products have been known for many decades to possess anthelmintic properties [5,6] and yet generally these have been inadequately researched and none have been taken to the market stage. One such group of potential anthelmintics is the cysteine proteinases found in fruits such as papaya, figs, kiwi fruits and pineapples. Laboratory experiments have demonstrated clearly that these compounds damage intestinal nematodes of rodents by a novel mechanism, targeting the cuticle which at first blisters, and then is disrupted and weakened sufficiently to enable the internal hydrostatic pressure to rupture the body wall and result in the disintegration of the worms [7]. Papaya latex, which contains high concentrations of four distinct cysteine proteinases [8], has been shown to have potent activity against worms of the stomach, small and large intestine in rodent model systems *in vivo*, causing dose-dependent reductions of worm burdens within safe treatment margins that resulted in little or no pathology [9-11]. Fig and papaya extracts have also been shown to reduce intestinal helminth infections in humans and in other monogastric animals including pigs [12,13].

Ruminants pose a formidable challenge for anthelmintic therapy, because after oral drenching the drugs pass first into the rumen, where their concentrations are greatly diluted and where they may reside for some time before gaining access to the abomasum and remaining sections of the intestinal tract [14]. Moreover the microbial and protozoal fauna of the rumen can cause drug efficacy to deteriorate. Indeed, papaya latex has previously been reported to have no efficacy against *Haemonchus contortus *in sheep [15] and to be highly toxic in ruminants [16].

Nevertheless, since papaya latex clearly has anthelmintic activity in monogastric animals, we were interested in reassessing the efficacy of papaya latex in sheep infected with nematodes. *H. contortus *was selected, because economically this is perhaps the most important parasite of sheep globally, responsible for major losses to the sheep industry [17]. We also assessed efficacy against *Trichostrongylus colubriformis*, which resides in the mucosa of the small intestine and which therefore is less exposed to compounds passing through the gut. In this paper we report the results of two experiments conducted in groups of lambs sufficient for robust statistical evaluation. These studies were aimed at determining the dose-dependence of four treatments at 24-hour intervals, the efficacy of a single dose, and the impact of neutralising abomasal acidity on the efficacy of papaya latex.

## Materials and methods

### Animals

In both experiments Greyface crosses [blueface Leicester tups on Scottish blackface ewes] were used. They were bred, raised and housed under conditions to minimise parasite contamination at the Moredun Research Institute animal holding facility. Each animal was screened for the presence of worms by faecal egg count examination prior to the start of the experimental infection. The sheep were approximately 4-5 months-old at the time that they were infected.

### Parasites

The anthelmintic susceptible isolates of *H. contortus *(MHco3; [18]) and *T. colubriformis *(MTco1; unpublished data) were used in these trials.

### Papaya latex

Four kg of *Carica papaya *spray-dried latex (Enzymase, P1) were dissolved in 12 litres of water. The preparation was centrifuged at 17,700 × *g *and the pellet was discarded. The supernatant was concentrated to a third of its original volume by placing in dialysis tubing [MW cut-off 3,500 (SpectraPor 45 mm diam.)] over polyethylene glycol 20,000. The concentrated material was aliquoted into individual containers and freeze-dried ready for reconstitution and oral delivery. Throughout the paper we refer to this preparation as the papaya latex supernatant (PLS).

At each step of the purification the molar concentration of active cysteine proteinase was assessed by titration with L-*trans*-epoxysuccinyl-leucylamido (4-guanidino)- butane (E64, Sigma-Aldrich) [19] with 4 mM L-cysteine as a reducing agent. The starting amount was 70 mmols of active enzyme in 4 kg of spray-dried latex, and during purification 41 mmols were retained in the freeze-dried supernatant. The individual aliquots of freeze-dried preparation contained 47, 117 and 234 μmol active cysteine proteinase by E-64 titration, these being the individual doses used for Experiment 1 (below). Throughout, once aliquoted and frozen, there was no deterioration of the enzyme activity and values derived before and after experiments were very similar.

### Haematocrit measurements

Venous blood was collected by jugular venepuncture into 10 ml heparinised vacutainer tubes (Becton Dickinson vacutainers systems) for microhaematocrit analysis expressed as percentage packed cell volume (PCV).

### Faecal egg counts

Faecal samples were taken *per rectum *throughout the course of the trial and faecal egg counts (FEC) were conducted on the material using a modification of the technique described by Jackson [20]. Total faecal egg counts were differentiated into *Haemonchus *and *Trichostrongylus *using previously described methodologies [13,14]. In brief the eggs from one gram of faeces from all individuals in each group were pooled and stained with peanut agglutinin (PNA) lectin. The PNA lectin binds preferentially to the carbohydrates on the egg shell of *H. contortus *[21,22].

### Necropsy and worm counts

All of the animals were necropsied on day 35 post infection ( p.i.) using the post mortem and worm recovery methods previously described [23]. The total worm burdens from the abomasa and small intestines were estimated from a sub-sample of the washings and saline digests. Ten percent subsamples (500 ml) were taken and stored.

Two percent aliquots were examined at x40 using a stereo microscope. The data from the washings and saline digests were pooled to provide total worm burden data. Recovered worms were sexed, staged and speciated [24].

### Cimetidine treatment

The antacid cimetidine (from the Pharmacy at the Queen's Medical Centre, Nottingham) (3.2 g in 50 ml water) was administered 20 mins before PLS in animals that received both treatments.

## Ethical approval

All experimental procedures described in this manuscript were examined and approved by the Moredun Research Institute Experiments and Ethics Committee and were conducted under approved British Home Office licenses in accordance with the Animals (Scientific Procedures) Act of 1986.

### Experimental design

Two experiments were conducted. The first experiment was designed to determine the efficacy of different amounts of PLS administered on 4 consecutive days on *H. contortus *and *T. colubriformis *faecal egg counts and worm burdens (dose-response experiment). The second experiment was split into three different sections. The first of these was to confirm any dose-dependent effect of PLS, the second investigated the efficacy of a single dose compared to 4 repeat doses of PLS, and the third examined any possible effect of an antacid, cimetidine, and additive or synergistic effects of the co-administration of cimetidine, with PLS.

For both Experiments 1 (*n *= 20) and 2 (*n *= 30) lambs were each infected with 5,000 infective larvae (L3) of *H. contortus *and 10,000 *T. colubriformis *L_3 _on day 0. The animals were randomly separated into groups of five. Treatments started on day 28 p.i., and for 3 consecutive days thereafter, aliquots of PLS preparations and cimetidine, as appropriate, were reconstituted with 50 ml of water and delivered orally by gavage. Animals were euthanased on day 35 p.i. unless otherwise stated.

### Experiment 1

The following treatment groups comprised Experiment 1:

Group 1 - water only, once daily for four days

Group 2 - 47 μmol active cysteine proteinase, once daily for four days

Group 3 - 117 μmol active cysteine proteinase, once daily for four days

Group 4 - 234 μmol active cysteine proteinase, once daily for three days

Note: Group 4 animals only had 3 treatments and were slaughtered on day 32 p.i.

FEC were conducted on days 21, 23, 25, 28, 29, 30, 31, 32 and 35 p.i. and on each occasion, except day 32, eggs were differentiated as described above.

### Experiment 2

This experiment comprised the following groups:

Group 1 - water only, once daily for four days

Group 2 - 50 μmol active cysteine proteinase, once daily for four days

Group 3 - 100 μmol active cysteine proteinase, once daily for four days

Group 4 - 100 μmol active cysteine proteinase, single treatment

Group 5 - cimetidine 3.2 g, once daily for four days

Group 6 - cimetidine 3.2 g then 50 μmol active cysteine proteinase, once daily for four days

FEC were conducted and eggs were differentiated as described above.

### Pathology

Immediately after euthanasia, all the carcasses were examined for any gross lesion and samples from the brain (internal capsule, hippocampus and midbrain) liver, kidney, lung and rumen were collected and placed into 10% formal saline. After fixation for five days, the samples were processed for histopathology by standard procedures for Haematoxylin and Eosin staining [25].

### Statistical analysis

Data are presented as arithmetic means and also as Log means [for FEC these were log_10_(EPG+25) and for worm counts log_10_(total worms +10)] because FEC and worm burdens are typically overdispersed in animals, and these transformations have been found in the past to best stabilise variance. Changes over time in FEC were carried out on log_10_(EPG+25) transformed data and analysed by repeated measures GLM (rmGLM) in SPSS (version 16.0.0 for Windows). If the assumptions of sphericity were not met, as assessed by Mauchley's test, we used the Huynh-Feldt adjustment to the degrees of freedom to err on the side of caution.

Worm counts were analysed by non-parametric tests, the Mann-Whitney *U *test for 2 group comparisons, the Kruskal-Wallis test for larger group treatment effects, and correlational relationships were examined with Spearman's rank order test, all using the software package SPSS (version 16.0.0 for Windows). For testing specific hypotheses we used the non-parametric Jonckhere-Terpstra test and for analyzing a 2 × 2 design the 2-way non-parametric ANOVA, both in the software package "Asking Questions in Biology", based on Barnard et al [26] and on Meddis [27].

## Results

### Experiment 1 - Dose-response effects of papaya latex supernatant on *H. contortus *and *T. colubriformis*

**Figure 1:**
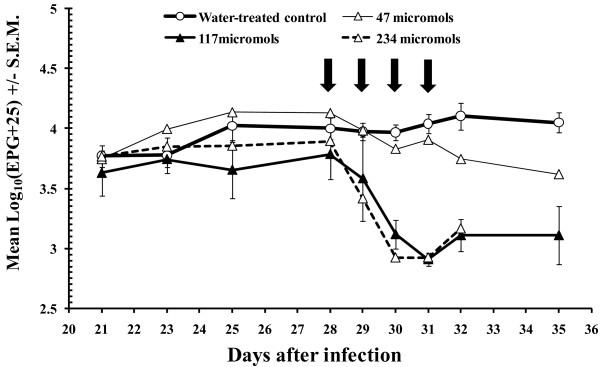
**Faecal egg counts during the course of Experiment 1**. Mean group values based on log_10 _(EPG+25) transformed data (± S.E.M.) with 5 animals per group. The bold vertical arrows show the days on which treatment was administered, the first dose being given on day 28 p.i.

In the control group treated with water, mean undifferentiated FEC values (Figure 1) were fairly steady throughout the period of observation, with arithmetic means varying from 6349 (±1182; standard error of the mean) on day 21 p.i. to 12010 (±2247) on day 35 p.i. with a peak count of 14143 (±2875) on day 31 p.i. As can be seen from Figure 1, undifferentiated FEC in all three groups treated with PLS showed some reduction in the days after treatment in contrast to the water-treated control group which rose marginally. In relation to day 28, just prior to treatment, and based on arithmetic means, FEC rose in the control group on day 35 by 12.0%, fell in Group 2 (47 μmol of PLS) by 66.4% and by 74.8% in Group 3 (117 μmol of PLS). In Group 4 (234 μmol of PLS) FEC fell on day 32 by 79.4%.

Group 4 (receiving the highest dose of PLS) was culled earlier on day 32 p.i. so that statistical analysis of FEC was based only on the first three groups, and this indicated that there was a significant main effect of treatment (between subjects effect, *F*_2,10 _= 7.95, *p *= 0.009). There was also a significant divergence between the groups in FEC over time (within subjects test, 2-way interaction time * treatment, *F*_13.8, 69.0 _= 7.43, *p *< 0.001).

**Figure 2:**
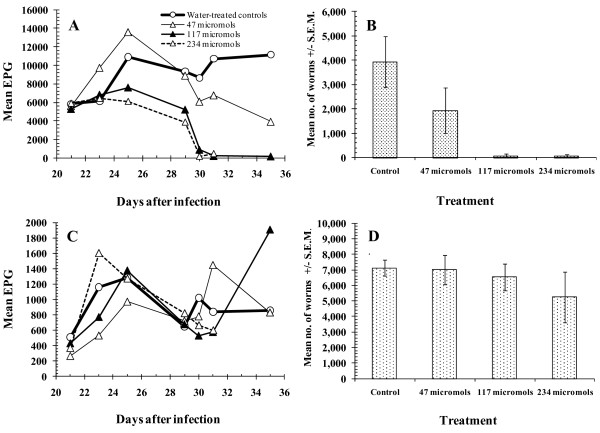
**Faecal egg counts (FEC) and worm burdens for *H. contortus *and *T. colubriformis *in Experiment 1**. **A**, FEC attributable to *H. contortus*, based on differential counts on pooled faeces at each time point, except day 32 p.i., when differential egg counts were not conducted; **B**, *H. contortus *worm burdens; **C**, FEC attributable to *T. colubriformis *based on differential counts, as above; **D**, *T. colubriformis *worm burdens. For details of treatments, differential counts and statistical analysis see text.

As expected, differential FEC showed that the majority of the eggs were from *H. contortus*, and that it was this species that was primarily affected by treatment (Figure 2A; note that no differential egg counts were conducted on day 32). As in the total FEC, there was a marked drop after treatment that was dose-related, and in the groups given the highest treatments, there was no subsequent rise between day 31 (last day of treatment) and autopsy on day 35.

The number of *T. colubriformis *eggs in faeces dropped during the treatment period, but then picked up soon afterwards in most groups (Figure 2C), increasing by d35 even in the animals treated with 117 μmol of active enzyme. However, in group 4, treated with 234 μmol, FEC were still low on d31 compared to earlier days, a day before these animals were culled.

In line with the drop in FEC with treatment, the two highest doses of PLS cleared *H. contortus *worms almost entirely from the abomasa (Figure 2B). Treatment with 117 μmol and 47 μmol resulted in 98.1% and 51.0% reduction in worm burdens, respectively. There was a highly significant effect of treatment (Kruskal-Wallis test, *χ*^2^_3 _= 11.57, *P *= 0.009 across all 4 treatment groups), that was dose-related (*r_s _*= -0.773, *n *= 19, *p *< 0.001).

There was no significant effect of treatment on *T. colubriformis *worm counts (Figure 2D) (Kruskal-Wallis test, *χ*^2^_3 _= 0.55, *p *= NS across all 4 groups). However, the data in Figure 2D suggest that *T. colubriformis *worm counts may have been lower in the group receiving the highest dose of PLS (log mean 26.1% lower relative to the control group) but comparison of the worm counts in this group (Mann-Whitney *U *test) with the water-treated control group gave *z *= -0.522, and *p *= NS. The lower mean count was attributable to two animals that had low counts (600 and 2200 worms), below the control range (5600-8700), the other 3 being well within and even above the control range (6950-9100).

### Haematocrits

Table 1 shows that PCV were highest in the animals treated with the highest doses of PLS, with a significant difference between treatment groups (*χ*^2^_3 _= 9.4, *p *= 0.025). This suggests a dose-response curative effect on haemonchosis-induced anaemia in the animals.

**Table 1:** Arithmetic mean packed cell volume (±SEM) of sheep in treatment groups on day 31 post infection

Treatment	Packed cell volume (SEM)
Water-treated control	21.5 (2.4) *n *= 5
47 μmol active proteinase	20.3 (1.6) *n *= 5
117 μmol active proteinase	27.9 (1.2) *n *= 4
234 μmol active proteinase	27.4 (1.7) *n *= 5

### Pathological examination

No gross lesions were appreciated in any of the sheep during the post-mortem studies.

Histologically, two animals showed mild focal interstitial inflammatory mononuclear infiltrates in liver, lung or rumen. There was a focal infiltration of a few mononuclear cells in the alveolar wall of one animal (4636G; 234 μmol group). Only one sheep (4634G; 234 μmol group) showed lesion in more than one organ: focal mild infiltration of mononuclear cells in the lamina propria of the ruminal papillae and mild hypercullarity of portal spaces in the liver.

### Experiment 2

#### Dose-dependence of the effect of PLS given over 4 days

**Figure 3:**
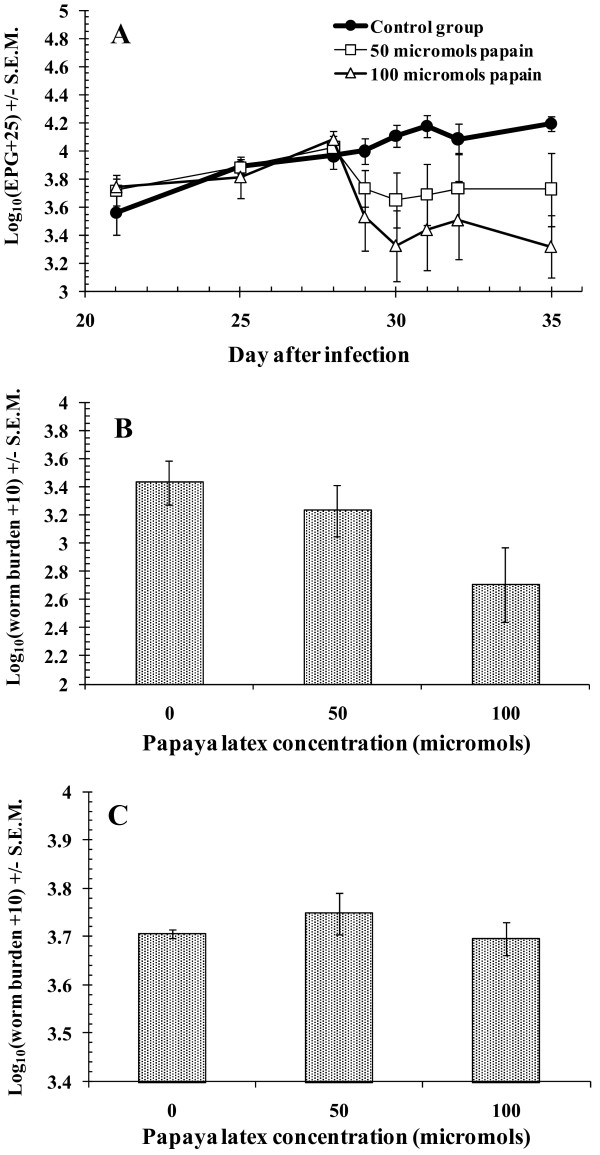
**Total FEC and individual worm burdens in sheep treated with different doses of PLS in Experiment 2**. **A**, Total FEC. The data are mean group values based on log_10_ (EPG+25) transformed data (± S.E.M.) with 5 animals per group. Repeated treatment was administered, the first dose being given on day 28 p.i. and the last on day 31; **B**, *H. contortus *worm burdens; **C**, *T. colubriformis *worm burdens.

As Figure 3A shows, treatment with PLS resulted in an immediate fall in FEC, and this reduction was more marked in the sheep receiving the higher dose (100 μmol active enzyme). The divergence between treatment groups was significant [rmGLM on log_10_(EPG+25) transformed data, within subjects test, interaction between time and treatment, *F*_5.4, 32.5 _= 3.63, *p *= 0.009]. There was a significant negative correlation between dose of active enzyme and EPGs on d35, the final day of the experiment (*r_s _*= -0.66, *n *= 15, *p *= 0.007).

Based on the highly significant dose-response found in Experiment 1, the hypothesis that *H. contortus *worm counts should be highest in the water-treated control group (group 1), intermediate in the animals given 50 μmol of PLS (group 2) and lowest in those treated with 100 μmol (group 3), was tested. The results are illustrated in Figure 3B, where it can be seen that worm counts declined with increasing dose of treatment [non-parametric Jonckhere-Terpstra test *z *= 2.02 ( *p *= 0.0218)]. Based on arithmetic mean worm burdens, and compared to the water-treated control group (4130 ± 1030) worm burdens fell by 43.6% in the group treated with 50 μmol of PLS for 4 days (2330 ± 832) and by 79.2% (860 ± 355) in those given 100 μmol. There was a significant negative correlation between worm counts and increasing doses of PLS (Spearman's one-tailed test *r_s _*= -0.539, *p *= 0.019). As in Experiment 1, and as illustrated in Figure 3C, *T. colubriformis *worm burdens did not decline with increasing dose of PLS. These results confirm the findings of Experiment 1, namely that treatment with PLS reduced *H. contortus *worm burdens in a dose-dependent manner but was without effect on *T. colubriformis*.

#### Comparison of repeated administration of PLS with a single dose

**Figure 4:**
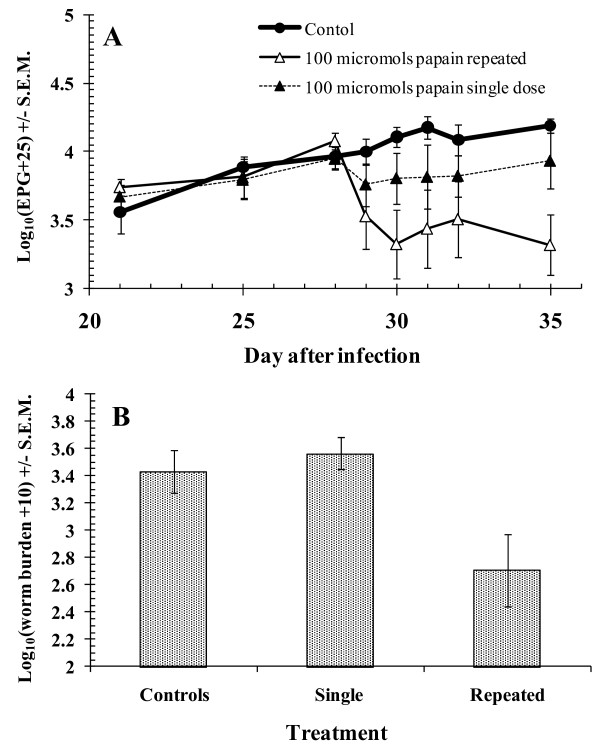
**Comparison of a single dose of 100 μmol PLS with 4 daily doses**. **A**, Total faecal egg counts; **B**, *H. contortus *worm burdens.

100 μmol of active cysteine proteinase administered as a single dose was compared with the same dose administered on 4 consecutive days and with a water control. Analysis of egg counts by rmGLM [duration of PLS treatment*time, on log_10_(EPG+25) transformed data, within subjects test] gave *F*_6.0, 35.9 _= 5.03 (*p *= 0.001) when the water-treated control group was included, indicating that FEC had diverged significantly with time between the 3 groups (Figure 4A). However, confining the analysis to the two infected groups to ascertain whether repeated administration was more effective than a single dose treatment gave only a borderline significance (treatment*time interaction, *F*_2.4, 19.3 _= 3.21, *p *= 0.053) and there was no significant difference between the single dose treatment and the control group (post-hoc test, treatment*time interaction, *F*_1,8_, = 1.26, *p *= 0.3). The main effect of treatment was not significant in any of these combinations.

Sheep that had received 4 doses of PLS had fewer *H. contortus *worms compared to those that had received the single treatment (2-tailed Mann-Whitney *U *test, *z *= -2.4, *p *= 0.016) (Figure 4B). However, there was no evidence for a reduction in worm burden with a single treatment. The animals given a single dose of 100 μmol of PLS had a worm burden with a higher arithmetic mean (4080 ± 716 worms) than the water-treated controls (3500 ± 1335) (Figure 4B). In respect of *T. colubriformis *worm burdens, the group given a single dose of PLS (group 4) had a lower arithmetic mean (4590 ± 429) than the water-treated control group (group 1; 5070 ± 106) and the repeatedly treated group (group 3; 5020 ± 375), but there was no significant difference between treated groups (Mann Whitney *U *test, group 3 vs group 4, *z *= -0.73, *p *= NS).

#### Effect of co-administration of an antacid and PLS

**Figure 5:**
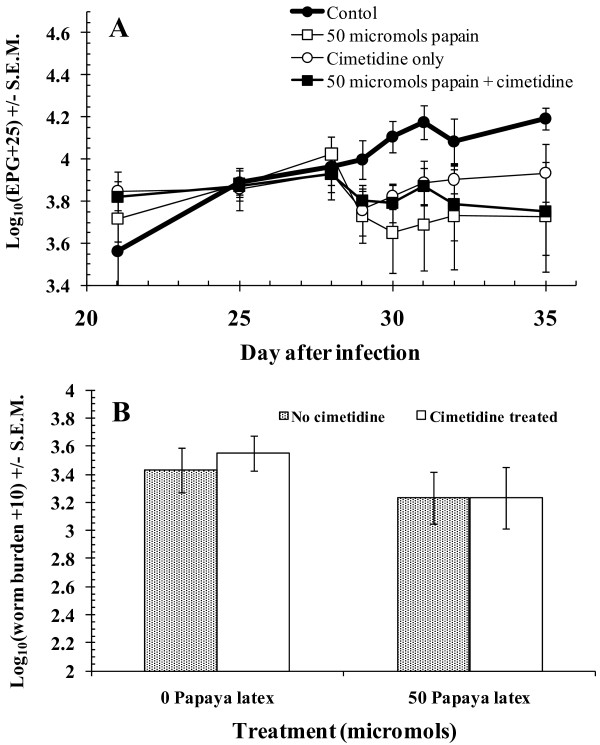
**Effect of cimetidine and PLS, administered separately or in combination, on total faecal egg counts and *H. contortus *worm burdens**. **A**, Total FEC; **B**, *H. contortus *worm burdens. Cimetidine (3.2 g) and PLS (50 μmol) were administered in isolation or in combination, on 4 consecutive days. In the latter case cimetidine was administered 20 min before PLS.

The co-administration of an antacid, cimetidine, with papaya latex has been shown to greatly increase efficacy against a murine stomach nematode [9], presumably because of the weak activity of the papaya proteinases at pH values ≤ 4.0 [28,29]. It has also been reported that cimetidine had an independent anthelmintic effect when administered directly into the abomasum [30]. We therefore decided to test the effect of cimetidine when administered orally, and investigate any additive or synergistic effect of cimetidine co-administered with a sub-optimal dose of PLS (50 μmol active cysteine proteinase). The FEC data are shown in Figure 5A. The summary data suggest that treatment with 50 μmol of active enzyme caused a rapid, sustained drop in FEC, relative to the control group. However, analysis by 2-way rmGLM with PLS treatment and cimetidine treatment, as factors and time as the within subjects factor, indicated that the divergence of FEC between treatment groups was of borderline significance only [PLS treatment *time, on log_10_(EPG+25) transformed data, within subjects test, *F*_3.5, 56.4 _= 2.589, *p *= 0.053]. There were no other significant terms in the full factorial model, and *post hoc *tests on individual days after the start of treatment did not provide support for any significant differences between treatment groups. The analysis therefore indicated that there was no enhancement of the anthelmintic activity when PLS was administered with the antacid cimetidine, and no independent anthelmintic effect of cimetidine when administered orally alone.

The results of worm counts of *H. contortus *are shown in Figure 5B. The data were analyzed by a 2-way non-parametric ANOVA. The two groups treated with PLS had marginally lower mean worm burdens (arithmetic means for group 2 = 2330 (±833) and group 6 = 2530 (±954), compared with group 1 = 3500 (±1335) and group 5 = 4130 (±1030), but at this dose of enzyme activity (50 μmol), there was no significant reduction in worm burdens, although this was just outside the cut-off (*z *= 1.36, *p *= 0.087). There was no enhancement of the anthelmintic effect in the animals co-administered cimetidine and PLS, and there was no overall effect of cimetidine on worm burdens.

For *T. colubriformis *there was no effect of PLS or of cimetidine, given separately or in combination (worm burdens in control group 1 = 5070 ± 10.6, and in group 2 treated with 50 μmol = 5700 ± 596; the cimetidine-treated group 5 had 5040 ± 358 worms and that given both 50 μmol of PLS and cimetidine (group 6) had 5270 ± 362 worms).

## Discussion

The results of the present study demonstrate for the first time that PLS possesses potent anthelmintic activity capable of clearing the adult parasitic nematode *H. contortus *from the sheep abomasum. The lack of efficacy of a single dose compared with the use of 4 daily doses suggests that, following dilution in the rumen, the enzymes require prolonged contact time with the worms in order to prove effective. Our results contrast with an earlier report of high toxicity of papaya latex in ruminants [16]. Although two animals showed histological lesions, they were mild and scant, and therefore considered not significant or related to diets or treatments administered to the animals. The presence of mild inflammatory infiltrates in the digestive system, liver or lungs is common in ruminants reared in outdoors or semi-outdoor conditions and we therefore consider them to be non-specific.

The *in vivo *anthelmintic efficacy of crude papaya latex against murine nematodes residing in various parts of the GI tract, has been reported previously [9-11]. However, in order to be able to scale up sufficiently to treat ruminants it was necessary to find a way to concentrate the active principles, the papaya cysteine proteinases found in the latex. A simple procedure of separation and concentration of the supernatant fraction allowed this, and at the same time may have removed contaminants that are toxic in ruminants. In our experiments, no toxic effects were detected with crude latex at effective anthelmintic doses in mice [11] nor was there any sign of toxicity in a pilot experiment where sheep were treated with crude latex preparations by gavage (results not shown). It is possible to monitor accurately the molar amount of active cysteine proteinase by the use of an active-site titrant, E64, that binds irreversibly to the active site of the enzymes and blocks activity, on a 1:1 molar basis [19,31]. The animals were dosed on this basis, rather than on the weight of the crude latex. Enzyme activity in papaya latex varies depending on the method of its preparation as well as other factors, and a highly active spray-dried preparation that is available in bulk and is employed in many manufacturing processes http://www.enzymase.com/production/process.htm was used as our starting material.

In our experiments, the effect of PLS was dose-dependent, consistent between experiments and enhanced by repeated dosing over 4 days, but not by co-treating with antacid (unlike earlier observations using murine stomach nematodes [9]). One reason for the lack of effect of an antacid may be the reported impact of nematode infection on abomasal pH. Infection with *Teladorsagia *(*Ostertagia) circumcincta *or *H. contortus *may lead to a rise in abomasal pH from as low as pH 2.5 up to approximately 6.5-7.0 [32,33], which is close to the pH optimum of the papaya cysteine proteinases, and therefore there was little scope for cimetidine to alter pH further. In our experiments abomasal pH was not monitored because this would have necessitated an abomasal fistula for the purpose and ethical approval was not granted. The increase in abomasal pH in infected animals suggests that the enzymes would only remain active in the abomasa in the presence of an infection, and that in animals with no or low worm burdens the abomasal pH would inhibit enzyme activity, thus preventing any unwanted side-effects on healthy animals.

The location of *T. colubriformis *in the mucosa, rather than the lumen, of the small intestine may explain its resistance *in vivo*. It is relevant that other species of intestinal nematodes that have temporary developmental stages in the tissues of their hosts are not affected by plant cysteine proteinases during the phases of their life cycles when they are actually in the mucosa as opposed to the intestinal lumen [10]. Given that we found no loss of *T. colubriformis *adult worms, and that we have observed similar *in vitro *damage on *T. colubriformis *(unpublished work) to that reported by us on other nematodes, lack of direct and prolonged access to the surface cuticle of *T. colubriformis *is the most likely explanation for the failure of PLS to show anthelmintic activity against this mucosal burrowing nematode. By tunnelling through the mucosa *T. colubriformis *may escape exposure to the direct effects of the cysteine proteinases as these pass down the gut.

The anthelmintic mechanism of action of papaya latex cysteine proteinases has been documented both *in vitro *and *in vivo *in rodents. The enzymes attack as-yet unknown protein targets in the cuticle, causing weakening of the cuticle, blistering and rupture, the release of internal tissues leading to the death of the worm. This property is not limited to the enzymes from papaya but appears to be a common property of papain homologues from pineapple, fig and from the latices of other plants [9-11,34-37].

There remains an urgent need to develop novel anthelmintics for controlling GI nematodes of domestic animals, as the available agents become less effective in the face of rapidly developing resistance by the parasites, and livestock agriculture suffers economic stresses as a consequence. We have suggested earlier [38] that resistance to naturally occurring cysteine proteinases may be very slow to develop, because the enzymes most likely target several of the constituent proteins that maintain cuticular structure and function, and therefore resistance to cysteine proteinases would be polygenic, rather than based on single point mutations as is sometimes the case for resistance to synthetic anthelmintics.

Although *T. colubriformis *proved to be unaffected by PLS in the current experiments, many other species of GI nematodes expose sufficient surface in the lumen of the gut to enable contact between the parasites and the enzymes. We predict that other, commercially important ruminant nematode species that reside in the gut lumen will prove to be susceptible to the action of papaya latex cysteine proteinases, thus endowing these enzymes with broad-spectrum activity.

## Competing interests

The authors declare that they have no competing interests.

## Authors' contributions

The work was conceived by DJBut, IRD, DJBart, HME, MCG, FJ and JMB. The experiments were conducted at the Moredun Institute with the animal trials under the direct supervision of FJ, DJBart, and YB, but all authors contributed to this phase in some degree. Partial purification and characterization of the proteinase preparations was carried out by DJBut and AL. The statistical analysis was conducted by JMB. The manuscript was written by JMB and DJBut and refined by all the remaining authors. All read and approved this final version of the ms.
